# Cervical Range of Motion Assessment through Inertial Technology: A Validity and Reliability Study

**DOI:** 10.3390/s23136013

**Published:** 2023-06-28

**Authors:** Martina Palmieri, Lucia Donno, Veronica Cimolin, Manuela Galli

**Affiliations:** 1 Department of Electronics, Information and Bioengineering, Politecnico di Milano, Piazza Leonardo da Vinci 32, 20133 Milan, Italy; martina.palmieri@mail.polimi.it (M.P.); lucia.donno@polimi.it (L.D.); manuela.galli@polimi.it (M.G.); 2 Istituto Auxologico Italiano, IRCCS, San Giuseppe Hospital, 28824 Piancavallo, Italy

**Keywords:** cervical spine, CROM, inertial sensor, validation, reliability

## Abstract

Inertial technology has spread widely for its comfortable use and adaptability to various motor tasks. The main objective of this study was to assess the validity of inertial measurements of the cervical spine range of motion (CROM) when compared to that of the optoelectronic system in a group of healthy individuals. A further aim of this study was to determine the optimal placement of the inertial sensor in terms of reliability of the measure, comparing measurements obtained from the same device placed at the second cervical vertebra (C2), the forehead (F) and the external occipital protuberance (EOP). Twenty healthy subjects were recruited and asked to perform flexion–extension, lateral bending, and axial rotation movements of the head. Outcome measurements of interest were CROM and mean angular velocities for each cervical movement. Results showed that inertial measurements have good reliability (0.75 < ICC < 0.9). Excellent reliability (ICC > 0.9) was found in both flexion and right lateral bending angles. All parameters extracted with EOP placement showed ICC > 0.62, while ICC < 0.5 was found in lateral bending mean angular velocities both for F and C2 placements. Therefore, the optimal sensor’s positioning emerged to be EOP. These results suggest that inertial technology could be useful and reliable for the evaluation of the CROM.

## 1. Introduction

The cervical spine is a remarkably complex segment of the vertebral column; it has a pivotal role in both influencing the global spine alignment and the pelvic tilt. Moreover, the cervical spine bears the load of the head, ensuring at the same time the widest range of motion (ROM) compared to the rest of the vertebral column. From an anatomical point of view, the cervical spine is divided into an axial region (C1 and C2 vertebrae) and a subaxial region (from C3 to C7 vertebrae). The seven vertebrae, which host ligamentous, capsular, tendinous, and muscular attachments, are separated by intervertebral disks with a “shock-absorbing” function.

As reported by Scheer et al. [1], the intricacies of the cervical region make it particularly susceptible to several disorders and dysfunctions, such that surgery could be warranted. Pathologies can affect the cervical mobility, interfering with the subject’s daily living with a non-negligible impact on the quality of life and work ability. Specifically, the global point prevalence of neck pain of a duration greater than 3 months increased by 21.1% from 2005 to 2015, probably due to the exponential increase in the hours spent in front of a screen while assuming an improper posture. Indeed, it is estimated that occupational ergonomic factors are considered responsible for 30.9% of the occurrences of neck pain and discomfort [2]. Actually, the World Health Organization ranked neck and back pain as the 12th leading cause of disability-adjusted life years (DALYs) in the world in 1990, and they moved up to 4th in 2015. Nowadays, different treatments are proposed to decrease pain and restore mobility. It is thus crucial to have efficient evaluation tools to choose the appropriate treatment and verify the benefits of this treatment. The anatomy of the cervical spine and the “resulting coupled movements” make the measurement of the cervical range of motion challenging. These coupled movements refer to the associate movement induced during movement targeted around one axis, e.g., around the medio-lateral axis, flexion is considered the primary movement, while simultaneous lateral bending and axial rotation are the associate movements. Hence, it becomes crucial to have easy access to tools that can provide a reliable evaluation of the cervical spine health status. In this sense, the measurement of the active cervical spine range of motion (aCROM or–more commonly–CROM) is considered a useful parameter to assess functional limitations, quantify the physical impairment, establish a treatment plan, and monitor the patient’s progress [3,4]. The most common way to clinically estimate cervical CROM is by quantifying angles and mean angular velocities of the following cervical movements: rotation, flexion–extension, and lateral bending.

While clinical questionnaires can give a subjective and qualitative evaluation, CROM provides a quantitative assessment of physical disability. Different methods have been proposed to measure CROM. Visual assessment, goniometers, measuring tape, radiography, and inclinometers are some of the most commonly used methods to evaluate CROM by healthcare professionals [5,6]. However, these tools are affected by their own intrinsic imperfections. Radiography’s main disadvantages are radiation exposure, as well as expense and time consumption [7]. Tape measures and goniometers, even if they are low-cost techniques, present a lack of accuracy and provide information in single planes and only for static positions [8]. As regards the goniometer, Tucci et al. [9] introduced an adapted gravity goniometer, and its reliability was tested and compared to the universal goniometer; they found a good reliability only for cervical extension measurement, while it was low for the other cervical movements. Electrogoniometers and inclinometers may offer solutions for more than one plane, as well as provide dynamic data; however, the physical design of such sensors can restrict motion [10]. Moreover, CROM is commonly analyzed during imposed movements in laboratory or clinical settings [4]. Therefore, this measurement may not be well-representative of cervical spine mobility in everyday life activities. In an attempt to overcome this limitation, a phone application was developed, although the CROM measurement during head rotation was not reliable due to magnetic interference [4,11]. In this sense, because of its high reliability and accuracy, the marker-based optoelectronic system, which is the gold standard for human movement analysis [10], would be a valuable option. However, due to its intrinsic complexity, this system is time-consuming, expensive, requires the need to wear few clothes, and requires well-trained operators [12]. Wearable inertial sensors are user-friendly tools that could allow clinicians and researchers to monitor and assess patients, reducing evaluation time and providing quantitative data on individuals’ functional limitations, according to the concept of ecological validity [13].

Nevertheless, before such technology can be used routinely in a clinical setting, its reliability and validity need to be assessed by comparing its performance with respect to a gold standard [10,14]. In this sense, the validity of the inertial sensor for assessing CROM has not been investigated. To the best of our knowledge, a previous study investigated the reliability of an inertial sensor system for assessing CROM and reported good to excellent reliability, pointing out that a different placement of the sensor could influence the reliability of data recording [15]. However, the study was conducted with a limited dataset and a small sample size. In another study [6], kinematic data of the cervical joints were simultaneously obtained using the inertial sensor, goniometer, and photographic measurements, demonstrating the high reliability and reasonable validity of the inertial sensor with respect to the other two methods. In the same study, authors concluded that an acceptable reliability is considered as a mean difference error of ±5°. To the best of the author’s knowledge, no similar conclusions could be found for mean angular velocities due to a lack of scientific research on the matter. However, no comparative studies using the gold standard technology, i.e., optoelectronic system, are present in the literature.

Hence, this work aims to assess the validity and reliability of the CROM measurement obtained by an inertial sensor in a group of healthy individuals when compared to that obtained by the optoelectronic system.

In addition, a further aim of this study is to test three different sensor placements to determine which one produces the most accurate measurements, therefore optimizing the accuracy of the data acquired.

## 2. Materials and Methods

### 2.1. Participants

Twenty healthy young adults (age: 28.6 ± 7.7 years, Body Mass Index, BMI: 18.5–24.9 kg/m^2^, 8 females, 12 males) were recruited for this study. The sample consisted of subjects without any neck pain, neck-related injuries, or any conditions of the cervical spine region in the last few years. Individuals with history of musculoskeletal or neurological disorders, pain, balance disorders, and other symptoms hampering the execution of the tests were excluded from the study.

All subjects were required to sign a written informed consent describing the experimental tests in detail. The study was conducted in accordance with the Declaration of Helsinki, and the protocol was approved by the Ethics Committee of Politecnico di Milano.

### 2.2. Data Acquisition

The inertial sensor was built with a triaxial accelerometer with multiple sensitivity levels (±2, ±4, ±8, ±16 g), a triaxial magnetometer 13 bit (±1200 μT), and a triaxial gyroscope 16 bit/axes with multiple sensitivity levels (±250, ±500, ±1000, ±2000°/s). The sensor works with an accelerometer frequency bandwidth ranging from 4 to 1000 Hz, a gyroscope bandwidth ranging from 4 to 8000 Hz, a magnetometer bandwidth up to 100 Hz, and sensor fusion up to 200 Hz. Acceleration data in the three directions (antero-posterior, medio-lateral and vertical) were acquired at 100 Hz frequency, transmitted via Bluetooth to a PC, and processed using the dedicated software (BAIOBIT 1.8 platform, Rivelo, Garbagnate Milanese (MI), Italy), which performs data acquisition, elaboration, reporting, and storage. The software used has a specific protocol capable of analyzing the CROM test and automatically generating a report.

Three different placements of the inertial sensor were investigated. Specifically, the device was placed on the second cervical vertebra (C2), on the forehead (F), and on the external occipital protuberance (EOP). To avoid possible slipping and consequent deviation from the nominal position, the inertial sensors were secured through an elastic band. However, it is possible that the reliability of the data recording could have been influenced by slippage, even if limited as far as possible, between the band and the hair/scalp.

**Figure 1:**
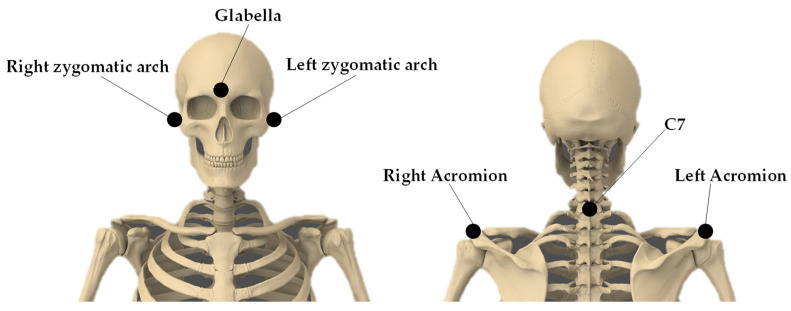
Marker set-up adopted for the study.

Then, six hemispherical infrared reflective markers were placed on the skin overlying the following specific anatomical landmarks (Figure 1): anteriorly to the tragus on the zygomatic arch (bilaterally), glabella, acromion (bilaterally) and the seventh cervical vertebra (C7) [16,17].

Markers trajectories were recorded by the optoelectronic system (SMART DX 6000, BTS Bioengineering SpA, Garbagnate Milanese (MI), Italy) and the dedicated software SMARTCapture (BTS Bioengineering SpA, Garbagnate Milanese (MI), Italy; version: 1.10.0470). Hence, each task was simultaneously recorded by both the inertial sensor and the optoelectronic system by the same operator, to limit the inter-individual errors and to ensure consistency.

Regarding the data collected via the optoelectronic system, a thorough calibration process was performed at the beginning of every acquisition day. Such process is composed of a first static calibration, used to set the global laboratory reference frame by means of three orthogonal wands, each one with a various number of spherical infrared markers (20 mm in diameter). The orientation of the laboratory reference frame had x-axis coinciding with the anterior-posterior direction, y-axis pointing upwards, and z-axis along the medio-lateral direction towards the right of the seated subject. Immediately after, the orthogonal triad of wands was removed and a single wand with 3 spherical infrared markers was properly moved by a trained operator to calibrate the acquisition volume.

**Figure 2:**
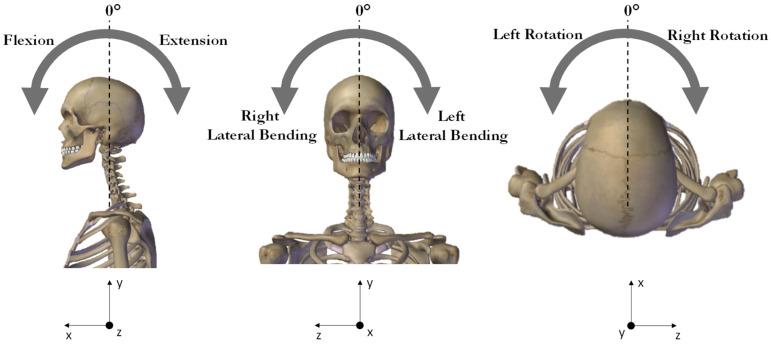
Schematic representation of the cervical movements. In the reference frame the x, y, and z axes indicate the anterior-posterior, proximal-distal, and medial lateral directions, respectively.

All measurements were performed with the subjects seated in an upright chair with their feet on the ground and keeping a proper posture to avoid any trunk contribution to the motor task. The individuals were instructed to start with open eyes focusing on a black circle positioned at the individual line of vision in front of him/her (starting position). After a brief familiarization period, participants were asked to perform maximal head flexion, extension, right axial rotation, left axial rotation, right lateral bending, and left lateral bending (Figure 2).

Each cervical movement was carefully described through specific operative instructions to provide a thorough understanding of the task as follows: rotation was explained as the movement required to draw an imaginary semi-circumference if the nose were to be a pencil, specifying that in doing so trunk and shoulders should be perfectly still. For flexion movement, the subject was asked to bring the chin towards the sternum, while for extension, to bring the top of the head towards the back, always focusing on moving just the cervical region. Finally, lateral bending was described as bringing the heart as close as possible to the tip of the ipsilateral shoulder, both left and right.

For limiting the fatigue effect, one minute of break was allowed between tasks. For each movement, three repetitions were performed, and participants were asked to complete the assessment at a self-selected speed.

The analysis was focused on each task on the CROM, computed as difference between the maximum angle value and the starting position value (neutral position), and the mean angular velocity. For data obtained from the inertial sensor, these parameters were automatically computed and displayed on the report. The software used was baiobit v.1.8 (Copyright © Rivelo Srl, 2021), which performs data acquisition, elaboration, and storage. The exact algorithm of data processing is private knowledge of the intellectual owner. For this study, data were collected using a sampling frequency of 100 Hz and transmitted via Bluetooth 3.0.

Regarding data acquired by the optoelectronic system, auto-labeling and tracking were performed via BTS^®^ TrackLab software (Copyright © 2023 BTS Bioengineering S.p.A. v.1.10.470.0). Each cervical movement task, as soon as it was completed by the subject, was visually inspected to manually label unmarked trajectories. When gaps were found, only those which were under 5 samples long were joined by means of linear interpolation. Otherwise, tasks were repeated. This process allowed for the correct 3D cervical movement reconstruction and the submission of data into the biomechanical application BTS^®^ SMARTAnalyzer (Copyright © 2023 BTS Bioengineering S.p.A v 1.10.470.0). Cervical reference system was obtained via the anatomical landmark of the glabella, left zygomatic arch, and right zygomatic arch. To avoid the possibility that a non-nominal orientation of the subject could make the movement less pure, thus distributing a portion of their intensity in the two non-principal directions, the reference system was corrected by rotating their vertical axis to align it with that of the laboratory reference system. The customized protocol used in this data analysis had the raw data preliminary pre-processed using a Butterworth fourth-order low pass filter (cut-off frequency 13 Hz).

Angular orientation and mean angular velocities for all three cervical movements were obtained based on the angle between the laboratory reference system and the cervical reference system. These parameters were computed using the software SMARTAnalyzer (BTS Bioengineering SpA, Garbagnate Milanese (MI), Italy; version: 1.10.470.0) starting from the three-dimensional trajectories of the markers according to the literature [16,17]. The midpoint between the two markers on left and right acromion processes was defined as the origin of a reference frame, parallel to the laboratory reference axes. The origin of the local reference frame of the head was defined as the midpoint between the markers over right and left zygomatic arch. The three markers on the head identified the head reference frame, while the three markers on the trunk were used for the identification of the trunk reference frame. The inclination of the head reference frame with respect to the trunk reference axes on the three anatomical planes provided the CROM during each movement.

### 2.3. Statistical Analysis

Data analysis was performed with IBM SPSS Statistics 27.0. Data normality was verified by applying the Shapiro–Wilk test; thus, the mean and the standard deviation were calculated for each parameter.

Based on a 2-way random effects analysis of variance, the ICCs (95% Cis) were computed separately for each of the three placements of the inertial sensor. ICC is commonly used to assess the consistency and reproducibility of objective measurements of the same quantity made by different observers or systems. ICC is a positive value that varies between 0 and 1. Data reliability was considered excellent if ICC > 0.90, good if 0.75 < ICC < 0.90, moderate reliability if 0.5 < ICC < 0.75 and finally poor if ICC < 0.5. In this case, ICC was used to check the correlation between the parameters obtained by the optoelectronic system and those by inertial sensor. A paired *t*-test was used to investigate even further the validity of the inertial sensor’s data. Bland–Altman plots are commonly used to assess the agreement between two or more instrumental measurement methods using a graphical representation of the differences. Thus, we included this test in our statistical analysis. This test was used to check whether the inertial sensor underestimated or overestimated parameters compared to the gold standard. The initial hypothesis was that the estimated parameters were within the Limits of Agreement (LoA). The LoA interval is established as the “mean difference × 1.96 × standard deviation”, according to the pre-established confidence interval (95%). For all the statistical tests, probabilities below 0.05 (*p* < 0.05) indicated rejection of the null hypothesis.

Finally, the accuracy of the measurement was assessed according to the following formula [Equation (1)].

(1) $$accuracy(\%)=\frac{measured\;value-actual\;value}{actual\;value}\cdot 100$$

where the actual value is the measurement value obtained by using the optoelectronic system, and the measured value is the measurement value of the inertial sensor.

## 3. Results

In Table 1, mean and standard deviation of all cervical mobility parameters and accuracy with respect to the gold standard measurements are reported when the inertial sensor is placed on EOP.

The paired *t*-test showed no statistically significant difference (*p* > 0.05) between data simultaneously collected from the optoelectronic system and the inertial sensor in any of the outcome measures of interest.

The accuracy presents good values for CROM measurements, except for the extension angle (12.62%). While regarding mean angular velocity measurements, the accuracy shows higher values, evidencing an overestimation of approximately 10°/s of the mean angular velocity during lateral bending tasks and almost 15°/s during axial rotation movements.

**Figure 3:**
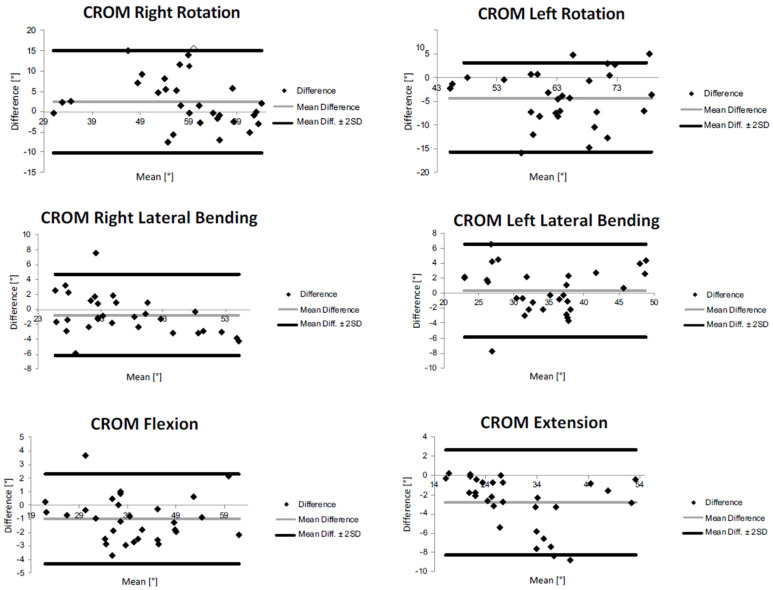
Bland−Altman plots of rotation, flexion, extension, and lateral bending CROMs for external occipital protuberance (EOP) sensor’s placement.

**Figure 4:**
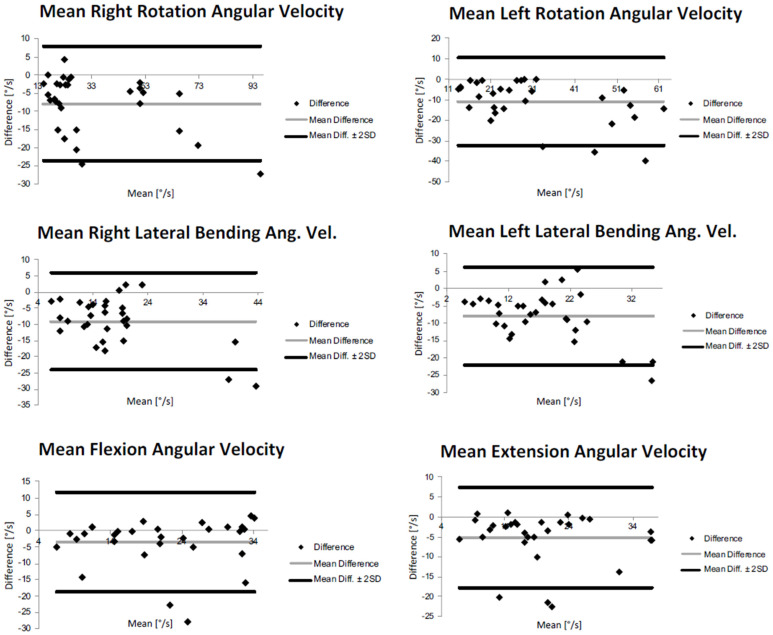
Bland−Altman plots of rotation, flexion, extension, and lateral bending mean angular velocities for external occipital protuberance (EOP) sensor’s placement.

The Bland–Altman plots calculated for the EOP placement for CROMs and mean angular velocities are depicted in Figure 3 and in Figure 4, respectively. The Bland–Altman is a scatterplot of the mean of the inertial system and optoelectronic system plotted against the difference between the two methods. In Figure 3, it can be noticed a wide interval of agreement (in some cases larger than 10°), especially for the right rotation. This could be probably due to both systematic and random errors during the acquisition protocol. However, in most cases, the range is narrower, and a high number of points is close to the mean difference. However, it is possible to observe that the Bland–Altman graphs display globally a good agreement between the two measurement systems demonstrating that most of the data were within the 95% consistency limit. In addition, the mean difference was close to zero in most of the measurements, and it was possible to observe that globally, there was an accordance between the two methods even if in each graph, some measurements were not in complete agreement. The outcomes indicated a bias smaller than 2° between the two measurement devices in determining cervical angles and of around 8°/s for mean angular velocities.

In Table 2, reliability analysis outcomes are summarized according to the three placements of the inertial sensor, using intraclass correlation coefficient (ICC) computed in the three configurations. An almost good reliability was found in the inertial data for all the CROMs measured (0.75 < ICC < 0.90).

In general, reliability outcomes were higher for the estimation of CROMs than mean angular velocities. In terms of CROMs quantification, ICC displayed values higher than 0.80 in all the three considered placements. However, the EOP placement seems to present the better values in terms of ICC. ICC > 0.90 was found for EOP location during all movements, except for left rotation, which presented an ICC value of 0.86. In the case of F placement, the ICCs were 0.92 and 0.95 during, respectively, the right rotation and extension tests while, from the C2, ICC values were 0.98 for flexion, 0.94 for extension and 0.92 and 0.91 for lateral bending CROMs measurements.

As regards the quantification of mean angular velocities, in C2 and F sensor placements, an ICC > 0.90 was obtained in flexion mean angular velocity and <0.50 during both lateral bending movements.

For what concerns the external occipital protuberance (EOP) sensor’s placement, ICC > 0.90 was found in flexion and right rotation mean angular velocities and ICC approximately close to 0.65 during both extension movements and lateral bending.

## 4. Discussion

In order to test whether an inertial sensor is capable of identifying the cervical spine range of motion (CROM), the maximal head and cervical spine flexion–extension, lateral bending, and axial rotation in a group of healthy individuals were analyzed simultaneously through an inertial sensor and optoelectronic system. The analysis of all CROMs measurements demonstrates the capability of the inertial sensor to extract cervical spine movement in agreement with the more complex and demanding gold standard optoelectronic system.

Our results exhibit a good agreement between the two systems in all the analyzed parameters. No statistical differences are found between the two systems, and good accuracy between the angular measurements, with the exception of cervical extension. As concerns the parameters related to angular velocity, the accuracy is not so satisfactory, evidencing an overestimation from 10 to 15°/s.

However, it is possible to observe that the Bland–Altman graphs display globally a good agreement between the two measurement systems in all the evaluated parameters, both CROMs and angular velocities.

In general, reliability appeared higher for the estimation of CROMs than for the mean angular velocities. Regarding CROMs quantification, the EOP placement seems to present the better values in terms of ICC, even if a general good/excellent reliability was found in all the three considered placements. The EOP location gave measurements with excellent reliability (ICC > 0.90) during all movements, except for left rotation, which is good (ICC = 0.86). In the case of F placement, the reliability resulted excellent during the extension and right rotation tests. The CROMs measurements of flexion, extension, and lateral bending, when recorded from the device placed on C2, showed excellent reliability. Hence, data collected by the device placed on EOP showed excellent reliability for the larger number of tasks compared to the other two placements. These reliability and validity outcomes confirm the established trend [3,6,18,19] in the field of human movement analysis, which employs more and more frequently inertial technology in day-to-day monitoring and assessment of the CROM, to perform evaluations that are both quicker and more adaptable to acquire data in a less constrained environment, such as an equipped gym or a race track or even just the patient’s home, still gaining, though, trustworthy and quantitative information.

As for the mean angular velocities, when the sensor was placed on C2 and F locations, an excellent reliability was obtained for the flexion task, while a low value of ICC (< 0.50) was recorded during right and left lateral bending movements, indicating poor reliability for these tasks. Regarding the sensor’s placement on the external occipital protuberance (EOP), the mean angular velocity during flexion and right rotation was measured with excellent reliability, whereas a moderate reliability (0.5 < ICC < 0.75) was obtained during both extension and lateral bending movements.

Interesting results were obtained in terms of different placements of the inertial sensor (second cervical vertebra vs. forehead vs. external occipital protuberance). While a global good reliability was found in the inertial data for all the CROMs measured (0.75 < ICC < 0.90), the results related to angular velocity revealed an appreciable difference in reliability between the different placements. Placements on the second cervical vertebra (C2) and forehead (F) showed poor reliability in the assessment of lateral bending mean angular velocity. Instead, when the device was placed on the external occipital protuberance (EOP), the resulting reliability was moderate in the case of lateral bending and extension angular mean velocity estimation, and it was excellent for the quantification of the mean angular velocity during both axial rotation and flexion. In reference to these results, it could be concluded that among the three placements of the inertial sensors analyzed in the present study, the one ensuring the most accurate measurements is the external occipital protuberance (EOP). Thus, with respect to the three tested sensor placements, the external occipital protuberance emerged as the most accurate. The second cervical vertebra could be less detectable as it is a relatively small landmark, and, on the other hand, the forehead is a quite broad region.

With respect to studies in the literature aiming at the validation of inertial technology [3,15,18,19,20], our study focused not only on validating the inertial sensor, providing a methodology based on a wearable inertial sensor that can be used easily in clinical settings for objective assessment of cervical mobility but also to investigate different sensor placements in order to see how different positions can influence the CROM measurement. Duc et al. [21] proposed a new evaluation tool based on the monitoring of cervical spine movement during daily activities using two inertial sensors in patients who underwent a cervical arthrodesis and in healthy controls. The proposed wearable system was revealed to be repeatable and valid for the evaluation of cervical spine function. They compared, in particular, the placements on the forehead and on external occipital protuberance, concluding that the former gives the most accurate measurement. However, this conclusion was achieved by only comparing values obtained between different placements of the inertial sensor, without any comparison with gold standard reference data. In another study, the same authors [22] aimed at assessing cervical spine mobility based on head and thorax kinematics measured with a wearable inertial system during active head movements (lateral bending, axial rotation, and flexion–extension) in 10 controls and 13 patients who had undergone an arthrodesis. They compared their data with the values obtained using an optoelectronic reference system. Movement patterns obtained by means of an inertial system showed excellent concurrent validity but presented slight differences in bias and dispersion. ROM obtained using an inertial sensor also showed some differences compared to the gold standard (mean difference < 5.7°). With respect to these studies, conducted on a small sample of individuals, our solution seems to have more concordance with the data obtained by the optoelectronic system in terms of ROMs; no comparison could be possible in terms of angular velocity as the previous studies were focused only on CROM and they did not assess other parameters. In addition, our study was conducted on a larger sample, even if composed of a young adult population.

The results obtained in this study showed that the proposed solution based on an inertial sensor could be suitable to quantify the cervical spine range of motion and angular velocity during fast and ample movements around the three axes of rotation, and they provided indications of the location for the inertial sensor (external occipital protuberance) allowing for the best reliability. The concordance and accuracy of the considered parameters with the gold standard are globally very good. Moreover, the inertial system and the dedicated report studied in this research were devised to be practical for clinical application as follows: the system is easy to wear, and the parameters are practically available immediately after recordings. Therefore, such a system provides clinicians with an accessible measurement system, in terms of cost and ease of use, for the objective evaluation of the cervical mobility in a clinical setting. Moreover, it is worthy of mention that not only the cost of the IMU is two orders of magnitude cheaper than the optoelectronic system, but also that the inertial sensor provides a different kind of analysis when compared to the optoelectronic system. Actually, the strength of optoelectronic systems lies in the quantification of the movement with a high degree of accuracy and precision. However, it can be used in the laboratory setting. The IMU overcomes the limitations of a traditional movement analysis laboratory since they were designed to collect daily living data and assess functional ability in the typical environment of the subject. This aspect is relevant since it is widely known that in a laboratory setting, patients feel they are being tested and are determined to maximize their performance, and therefore, the result obtained in a laboratory setting could not fully reflect the subjects’ abilities in daily life.

Hence, this IMU seems a valuable tool when simple and fast assessments are required, as in day hospital settings or rehabilitative contexts in which this technology can automatically provide a fast quantification of parameters useful for disease monitoring in shorter assessment time frames.

By now, this technology was validated on a young adult population; future research should be conducted on a pathological cohort that shows non-physiological trends of CROM; in this way, it could be possible to assess if inertial sensor measurements are repeatable and valid for the evaluation of cervical spine also in pathological conditions. In this way, it would be possible to assess the severity of motion limitation and level of effort in cervically involved patients to evaluate the follow-up performance during and after conservative or invasive interventions. This would allow us to further assess inertial measurements’ robustness.

## 5. Conclusions

The current study proposed and applied a method for estimating the cervical spine mobility using a single inertial sensor. This solution was revealed to be suitable for the quantification of cervical spine range of motion with a satisfactory accuracy and reliability in comparison with the gold standard technology for human movement. Among the three different placements of the device–second cervical vertebra, forehead, and external occipital protuberance–the latter offered the best reliability. In conclusion, the inertial technology could represent a low-cost, non-invasive, user-friendly, and flexible tool for a reliable evaluation of the cervical spine range of motion also in environments where the traditional three-dimensional movement analysis is not applicable. The proposed method seems to have a good repeatability and validity when measuring CROM and is an effective way to evaluate the cervical vertebral range of motion that may be used in clinical practice.

## Figures and Tables

**Table 1:** Mean and standard deviation of CROMs and mean angular velocities in right and left rotation, flexion, extension, and right and left lateral bending cervical movements.

	Optoelectronic System (Mean ± SD)	Inertial Sensor (Mean ± SD)	Accuracy (%)
CROM			
Right Rotation [°]	60.4 ± 11.6	60.4 ± 12.5	0.01
Left Rotation [°]	66 ± 10	63.4 ± 10.3	3.94
Flexion [°]	37.6 ± 15.6	36.7 ± 14.7	2.39
Extension [°]	31.7 ± 11.7	27.7 ± 9.7	12.62
Right Lateral Bending [°]	38.2 ± 9.2	38.4 ± 8.5	0.52
Left Lateral Bending [°]	35.2 ± 8.7	36.8 ± 9.2	4.55
Mean angular velocity			
Right Rotation [°/s]	37.9 ± 18.9	24.8 ± 14.6	34.56
Left Rotation [°/s]	37.6 ± 15.9	22.7 ± 19.3	39.63
Flexion [°/s]	21.9 ± 10.5	18.7 ± 9.6	14.61
Extension [°/s]	22.6 ± 9.8	18 ± 8.2	20.35
Right Lateral Bending [°/s]	22.5 ± 10	10.5 ± 7.7	53.33
Left Lateral Bending [°/s]	21.5 ± 8.6	10.2 ± 5.1	52.56

**Table 2:** Reliability analysis (intraclass correlation coefficient–ICC) results for CROMs and mean angular velocities relative to the inertial sensor placed on second cervical vertebra (C2), on the forehead (F), and on the external occipital protuberance (EOP).

	C2	F	EOP
CROM			
Right Rotation [°]	0.86	0.92	0.92
Left Rotation [°]	0.85	0.86	0.86
Flexion [°]	0.98	0.75	0.98
Extension [°]	0.94	0.95	0.92
Right Lateral Bending [°]	0.92	0.88	0.94
Left Lateral Bending [°]	0.91	0.84	0.93
Mean angular velocities			
Right Rotation [°/s]	0.77	0.6	0.91
Left Rotation [°/s]	0.72	0.44	0.77
Flexion [°/s]	0.93	0.95	0.92
Extension [°/s]	0.83	0.84	0.66
Right Lateral Bending [°/s]	0.34	0.28	0.62
Left Lateral Bending [°/s]	0.28	0.28	0.63

## Data Availability

Data available on request due to restrictions, e.g., privacy or ethical.
